# Antioxidative properties, phenolic compounds, and in vitro protective efficacy of multi‐herbal hydro‐alcoholic extracts of ginger, turmeric, and thyme against the toxicity of aflatoxin B_1_ on mouse macrophage RAW264.7 cell line

**DOI:** 10.1002/fsn3.4257

**Published:** 2024-08-20

**Authors:** Nina Nazdar, Ahmad Imani, Seyyed Meysam Abtahi Froushani, Mohsen Farzaneh, Kourosh Sarvi Moghanlou

**Affiliations:** ^1^ Department of Fisheries, Faculty of Natural Resources Urmia University Urmia Iran; ^2^ Department of Microbiology, Faculty of Veterinary Medicine Urmia University Urmia Iran; ^3^ Department of Agriculture Medicinal Plants and Drugs Research Institute, Shahid Beheshti University Tehran Iran

**Keywords:** cell culture, cell viability, HPLC, mycotoxin, plant extracts

## Abstract

Aflatoxin B_1_ (AFB_1_), the most potent toxic and carcinogenic secondary fungal metabolite, has frequently been reported in food/feed. Nowadays, herbal extracts are considered safe dietary additives to reduce the toxicity of such compounds. The protective capability of various combinations of hydro‐alcoholic extracts (HAEs) of ginger, turmeric, and Shirazi thyme, against the toxicity of AFB_1_ on the RAW264.7 cell line was investigated. The RAW264.7 cells were exposed to six different concentrations of AFB_1_ (0.09, 0.18, 0.37, 0.75, 1.5, and 3 μg mL^−1^) for 48 h to determine the IC_50_ of AFB_1_. AFB_1_ was estimated to have an IC_50_ of 1.5 μg mL^−1^ for RAW264.7 cells. Then, the cells were simultaneously incubated with 1.5 μg mL^−1^ AFB_1_ and the HAEs for 24 h. The HAEs significantly reduced the toxicity of AFB_1_ in RAW264.7 cells. HAE of Shirazi thyme showed the highest amount of total phenol content (TPC) and the highest DPPH• activity. In addition, a combination of ginger, turmeric, and Shirazi thyme extract showed the highest antioxidant activity. Rutin, quercetin, and apigenin were the main phenolic components of ginger HAE. A significantly positive correlation was observed between TPC of hydro‐alcoholic extract with ferric reducing antioxidant power (FRAP) and 3‐(4,5‐dimethylthiazol‐2‐yl)‐2,5‐diphenyltetrazolium bromide (MTT) values. Consequently, the simultaneous consumption of such extracts is recommended to protect the cells against dietary toxins.

## Graphical abstract

Aflatoxin B_1_ considerably affected mouse macrophage RAW264.7 cell line survival. Shirazi thyme HAE showed the highest DPPH‐free radical trapping activity. A significantly positive correlation was observed between the TPC of HAEs and FRAP or MTT.

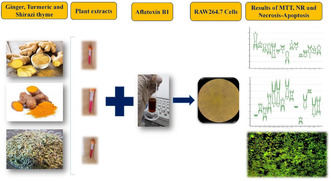

## 1 INTRODUCTION

Toxins, especially mycotoxins, found in various products, are considered a severe challenge for human and animal health (Kendra & Dyer, 2007). Because mycotoxins are carcinogenic, they may cause neurotoxicity and developmental abnormalities (Anater et al., 2016). Three fungal genera, including *Aspergillus*, *Fusarium*, and *Penicillium*, are mainly involved in the contamination of food/feed commodities with mycotoxins (Marin et al., 2013).

Aflatoxins (AFs), especially AFB_1_, are the most critical fungal toxins in terms of occurrence and toxicity; they are mainly produced by *Aspergillus* species (Kumar et al., 2017) and are classified as the most common natural carcinogenic compounds by the International Agency for Research on Cancer (IARC) and the US Food and Drug Administration (FDA) (WHO‐IARC, 1993). The aflatoxins might cause DNA damage and subsequently affect DNA repair pathways (Bhatnagar et al., 2006). The lethal dose (LD_50_) varies from 0.5 to 10 mg kg^−1^ for all types of AFs, depending on the sensitivity of the target animal (Shephard, 2008). AFB_1_ is converted into aflatoxin B1‐8,9‐epoxide (AFBO) by the action of cytochrome P450 in the liver and intestine. The AFBO has the capability of epoxide covalent bonding with macromolecules such as RNA, DNA, and proteins, which leads to genotoxicity when attached to DNA and cytotoxicity when attached to proteins and also leads to lipid peroxidation of the cell membrane. The AFBO binds to guanine residues in DNA and forms aflatoxin‐N7‐guanine, resulting in G to T transition mutations. It has been shown that any mutations in codon 249 of the tumor suppressor p53 gene might lead to hepatocellular carcinoma (HCC) in animals (Ahmed Adam et al., 2017).

Various environmental factors could result in oxidative stress and the surplus production of free radicals (Young & Woodside, 2001). Oxidative stress occurs in cells when the production of reactive oxygen species (ROS) and reactive nitrogen species (RNS) surpasses the antioxidative capacity of cells/bodies (Valko et al., 2007). The toxicity of AFs might be related to increased intracellular production of ROS such as superoxide anion, hydroxyl radical, and hydrogen peroxide (H_2_O_2_) following biotransformation of AFB_1_ by cytochrome P450 (Towner et al., 2003). Any exposure to aflatoxin might affect all cellular functions, including protein synthesis, cell apoptosis, etc. (Pang et al., 2020). AFB_1_ exposure could result in oxidative stress and finally the activation of multiple signaling pathways associated with the inflammatory response, which might bring about macrophage phagocytosis inhibition (Ma et al., 2021).

Inflammation is a pathophysiological cellular response that arises from the recruitment of local immune cells and the accumulation of plasma fluid (Sosa et al., 2002). The biological phenomenon of inflammation encompasses a multifaceted process that incorporates a series of distinct factors, such as prostaglandins, leukotrienes, and platelet‐activating factors (PAFs) (Tunon et al., 1995), which affect body health status. The macrophages play a crucial role in inflammatory disease by releasing nitric oxide (NO), prostaglandins, and cytokines (Eidi et al., 2018). The cyclooxygenases (COXs) are involved in synthesizing prostaglandins, prostacyclins, and thromboxane from arachidonic acid (Hebbes & Lambert, 2008). Nitric oxide mediates various biological events. Research has shown that inhibition of its synthesis may be helpful in treating inflammatory diseases (Terra et al., 2007). Furthermore, anti‐inflammatory compounds can inhibit prostaglandin synthesis by cyclooxygenases. A diverse range of steroidal and non‐steroidal drugs (NSAIDs) are used to combat inflammation. However, their many side effects have limited their long‐term administration (Yildirim et al., 2019).

Plants' secondary metabolites, including phenolic compounds, are generally regarded as safe (GRAS) (Smid & Gorris, 1999) with noticeable free radical inhibitory properties (Pandey et al., 2016). Metabolites are biodegradable and environmentally friendly, biologically safe, readily available, and cost‐effective; thus, their use has attracted worldwide attention (Vijayanandraj et al., 2014).

Ginger (*Zingiber Officinale*) belongs to the family of Zingiberaceae and is native to Southeast Asia (Singletary, 2010), and its rhizomes contain carbohydrates (50%–70%), lipids (3%–8%), phenolic acids, and terpenes (Mele, 2019). The anti‐inflammatory components, such as 6‐gingerol, 6‐shugval, and zeningerol, could reduce the production of inflammatory cytokines and chemokines through anti‐inflammatory, antioxidant, and anti‐serotonin activities (Mele, 2019). The plant extract with anti‐inflammatory properties showed inhibitory effects on COX and NF‐KappaB (κB) activity (Grzanna et al., 2005). Turmeric (*Curcuma longa* L.), also belongs to the ginger family (Zingiberaceae) (Wakte et al., 2011), while its rhizomes contain carbohydrates (69.4%), protein (6.3%), lipids (5.1%), and minerals (3.5%) (Prasad et al., 2014). Also, it contains curcuminoid compounds, including curcumin (77%), demethoxy curcumin (DMC; 17%), and bisdemethoxy curcumin (BDMC; 3%) (Goel et al., 2008). Curcumin presents various pharmacological implications attributed to its antioxidative, anti‐inflammatory, antimicrobial, and anti‐cancer effects (Kocaadam & Şanlier, 2017). Shirazi thyme (*Zataria multiflora* Boiss) belongs to the Lamiaceae family (Sharififar et al., 2017), and its essential oil is rich in carvacrol (Kavoosi et al., 2012). Shirazi thyme and carvacrol are used in managing asthma, chronic obstructive pulmonary disease (COPD), and as antioxidant and anti‐inflammatory medications (Mohebbati et al., 2018).

Macrophages are mononuclear cells that play an essential role in the body's innate and acquired immune systems. The toxicity of AFB_1_ on macrophages has been documented (Bruneau et al., 2012). In the present study, multiherbal hydro‐alcoholic extracts of ginger, turmeric, and Shirazi thyme were prepared. Their antioxidative properties, along with the contents of 18 phenolic compounds, were determined. To measure the protective effects of such extracts against the toxicity of AFB_1_, the RAW264.7 cell line was used. All analyses were carried out three times for each treatment/experiment. Finally, through MTT and NRU tests, and cellular morphology examination by fluorescence microscopy of cells, a multiherbal extract with the highest protective potency against the IC_50_ of AFB_1_ was identified.

## 2 MATERIALS AND METHODS

### 2.1 Preparation of HAEs of the herbs

To prepare the plant extracts, the rhizomes of ginger (*Z. officinale*) and turmeric (*C. longa*) and the aerial parts of Shirazi‐thyme were prepared, dried at room temperature and in darkness, and ground to powder. To prepare extracts, 200 g of the pulverized plants was extracted with 1000 mL of ethyl alcohol:double distilled water 50:50 mixture (v:v). The mixture was intermittently stirred for 48 h at room temperature. Afterward, the extract was obtained through overnight precipitation at 4°C in cylinder‐conical glassware and multiple centrifugations at 1000 ***g*** for about 2 h. Finally, the resultant extract was passed through a 0.2 μm filter and concentrated using a rotary evaporator apparatus at 45°C until complete dryness (Moosavi et al., 2020). The dried extract (1 g) was re‐solved in 100 mL double‐distilled water (final concentration 1%) for TPC determination, antioxidative properties, and HPLC studies. While, to re‐solve the dried extract for the follow on experiments on the cell line, 10 mL of Dulbecco's modified Eagle's medium (DMEM) culture medium was used to prepare the stock solution, which contained 6 mg of the dry extracts (concentration 0.06%).

### 2.2 Phytochemical characteristics of herbal extracts

#### 2.2.1 Determination of TPC

The measurement of TPC was conducted utilizing the Folin–Ciocalteu reagent (Slinkard & Singleton, 1977). 180 μL of double‐distilled water was added to 10 μL of each plant extract (1 g. 100 mL^−1^) obtained from resolving the dried plant extract (see Section 2.1). 1200 μL of Folin (10%) was mixed with the resultant extract, and after 5 min, sodium carbonate (7.5%) was added. The samples were subjected to a period of incubation of 30 min in darkness at room temperature. Finally, the absorbance was read by a spectrophotometer (Dynamic HALO DB‐20, UK) at 760 nm. The calibration curve was plotted using gallic acid as the standard, and TPC was reported as mg of gallic acid equivalent (GAE) g dry weight (DM)^−1^ of the extract.

### 2.3 HPLC‐PDA^1^ analysis of phenolic compounds

The HPLC‐UV analysis of the contents of 18 phenolic compounds, including gallic acid, 3,4‐dihydroxybenzoic acid, catechin, chlorogenic acid, vanillic acid, caffeic acid, 2,5‐dihydroxybenzoic acid, syringic acid, p‐coumaric acid, ferulic acid, chicoric acid, rutin, rosmarinic acid, salicylic acid, quercetin, cinamic acid, kaempferol, and apigenin, of each extract was done according to Ghaderi et al. (2019). The Waters 2695 Alliance HPLC system equipped with a PDA 996 detector and C18 column (25 cm × 4.6 mm Eurospher 100‐5) was used for quantification analysis of the phenolic compounds. The mobile phase included (A) methanol +0.02% trifluoroacetic acid (TFA) and (B) HPLC grade water+0.02% TFA, and ran at a flow rate of 0.5 mL min^−1^ under the gradient program for 60 min. The chromatograms were monitored at 200–400 nm wavelengths to identify each phenolic compound.

### 2.4 Determination of antioxidant properties

#### 2.4.1 Ferric‐reducing antioxidant power assay (FRAP) of the extracts

Antioxidant activity was also spectrophotometrically determined by ferric reducing power at 593 nm. 10 μL of aqueous solution of each dried plant extract (1 g. 100 mL^−1^) was mixed with 3 μL of FRAP solution. The mixture was incubated at 37°C for 30 min in the water bath. Iron chloride was used to plot the standard curve (Tomasina et al., 2012).

#### 2.4.2 DPPH• free radical scavenging activity

To measure antioxidant activity by the DPPH• scavenging method, 2000 μL of DPPH• solution (0.006 g of DPPH• in 150 mL of 80% methanol) was mixed with 10 μL of aqueous solution of each dried plant extract (1 g. 100 mL^−1^). The resulting solution was shaken and kept at room temperature for 30 min. The color of the mixture changed from dark violet to light violet and pink (Nakajima et al., 2004). Finally, its absorbance was spectrophotometrically recorded at 517 nm.

$$\text{Inhibition}\;\left(\%\right)=[({\mathrm{Abs}}_{\text{blank}}-{\mathrm{Abs}}_{\text{sample}})/{\mathrm{Abs}}_{\text{blank}}]\times 100$$

### 2.5 Cell culture

The RAW264.7 cell line was obtained from the Faculty of Veterinary Medicine of Urmia University cell bank. The cells were cultured in DMEM high glucose medium (Dulbecco's modified Eagle's medium) supplemented with 10% heat‐inactivated fetal bovine serum (56°C water bath, for 40 min), 1% pinstripe antibiotics (penicillin and streptomycin), and 2% L‐glutamine at 37°C, 5% CO_2_ (Robbe et al., 2015). To isolate cells from flasks, a solution containing lidocaine (4 mg mL^−1^) and EDTA (10 mM) was utilized (Robbe et al., 2015).

### 2.6 Determining the IC_50_ of AFB_1_ in the RAW264.7 cell line

According to Zhou et al. (2017), different concentrations of AFB_1_ including 0, 0.09, 0.18, 0.37, 0.75, 1.5, and 3 μg mL^−1^ were used. After 48 h of AFB_1_ exposure of the macrophage cells, cell viability and vitality were determined by 3‐(4,5‐dimethylthiazol‐2‐yl)‐2,5‐diphenyltetrazolium bromide (MTT) and lysosomal uptake of neutral red, a week cationic dye, assays, respectively (Shushtari & Froushani, 2017).

### 2.7 Determination of plant extracts safety for the RAW264.7 cell line

Viable 10 × 10^6^ cells were cultured in 96‐well microplates and incubated for 24 h with different concentrations of ginger, turmeric, and thyme HAEs (1.56, 3.12, 6.25, 12.5, 25 μg mL^−1^) (Suharty & Wahyuni, 2018). The viability of cells was determined by the MTT assay (Nemudzivhadi & Masoko, 2014). The safe concentration of the individual extracts was combined (Table 1), and the safety of multi‐herbal extracts was also accordingly assessed.

**TABLE 1:** Multi‐herbal extracts used for safety assessment on the RAW264.7 cell line.

Treatment	Label	Thyme (A) (μg mL^−1^)	Turmeric (T) (μg mL^−1^)	Ginger (Z) (μg mL^−1^)
A‐T (1)	Thyme + Turmeric	1.56	0.78	0
A‐T‐Z (1)	Thyme + Turmeric + Ginger	0.78	0.78	1.56
T‐Z (1)	Turmeric + Ginger	0	1.56	1.56
A‐Z (1)	Thyme + Ginger	0.78	0	3.12
A‐T‐Z (2)	Thyme + Turmeric + Ginger	1.56	1.56	1.56
A‐T‐Z (3)	Thyme + Turmeric + Ginger	1.56	0.78	3.12
A‐Z (2)	Thyme + Ginger	1.56	0	1.56
T	Turmeric extract	0	0.78	0
Z	Ginger extract	0	0	1.56
A‐T‐Z (4)	Thyme + Turmeric + Ginger	0.78	1.56	3.12
A‐T (2)	Thyme + Turmeric	0.78	1.56	0
A	Thyme plant extract	0.78	0	0
T‐Z (2)	Turmeric + Ginger	0	0.78	3.12

### 2.8 Assessment of cell‐protective properties of multi‐herbal extracts against IC_50_ AFB_1_ exposure

Various plant HAE combinations (Table 2) were included to elucidate their protective effects on AFB_1_‐exposed cells for 24 h. Finally, an MTT assay was carried out to determine cell viability (Skrzydlewski et al., 2022).

**TABLE 2:** Various experimental treatments, including different (multi)herbal extracts and AFB_1_, on the RAW264.7 cell line used in MTT and NRU assays.

Treatment	Thyme (A) (μg mL^−1^)	Turmeric (T) (μg mL^−1^)	Ginger (Z) (μg mL^−1^)
A‐T (1)	1.56	0.78	0
A‐T‐Z (1)	0.78	0.78	1.56
T‐Z (1)	0	1.56	1.56
A‐Z (1)	0.78	0	3.12
A‐T‐Z (2)	1.56	1.56	1.56
A‐T‐Z (3)	1.56	0.78	3.12
A‐Z (2)	1.56	0	1.56
T	0	0.78	0
Z	0	0	1.56
A‐T‐Z (4)	0.78	1.56	3.12
A‐T (2)	0.78	1.56	0
A	0.78	0	0
T‐Z (2)	0	0.78	3.12
Control	(Cell and AFB_1_)		
Blank	(Cell and culture medium)		

#### 2.8.1 MTT assay

The MTT assay measures the metabolic activity of cells to convert tetrazolium compounds to water‐insoluble formazan crystals by dehydrogenase enzymes in mitochondria. The formazan crystal is then dissolved in DMSO, and the resulting color solution was quantified by determining optical density at 540 nm (van Tonder et al., 2015). The cells were introduced into a 96‐well plate at 16 × 10^6^ cells mL^−1^, exposed to various concentrations of herbal extracts (Table 2), and/or IC_50_ AFB_1_ at 37°C for 48 h. Then, 10 μL of MTT reagent (5 mg mL^−1^ in PBS) was added. After a 4‐h incubation period at 37°C, the medium culture was removed, and the cells were exposed to 100 mL of DMSO. Finally, the optical density was measured at 540 nm (ELISA reader; Bio‐Rad, USA) (Forouharmehr et al., 2013).

#### 2.8.2 Neutral red uptake assay (NRU)

The neutral red uptake assay is based on the ability of living cells to uptake neutral red dye, which includes the following steps: (1) passive non‐ionic diffusion of the dye into cells; (2) accumulation of the dye in cell lysosomes; (3) extraction of the dye from living cells using an acidified ethanol solution; and (4) quantification of the solution by measuring the optical density at 540 nm (Skrzydlewski et al., 2022).

### 2.9 Assessment of apoptotic and necrotic cells using fluorescence microscopy

Acridine orange (AO) and propidium iodide (PI) are nuclear staining dyes. AO is absorbed by live and dead cells and would emit green fluorescence if it entered the double‐stranded nucleic acid (DNA) structure (Zhang et al., 1998). PI is a fluorescent nucleic acid stain that enters cells with low membrane integrity. The dye causes cells with disrupted membranes to glow red under fluorescence light. It has been shown that loss of membrane integrity is the main feature of necrotic cells (Attari et al., 2009). Cells were incubated with the dye solutions for 20 min at 37°C. Eventually, stained cells were observed under a fluorescence microscope. The microscopic analysis was carried out three times for each treatment/experimental group.

### 2.10 Statistical analysis

Analyzing data and drawing graphs were done using MedCalc 18.9.1 and Microsoft Office Excel 2013, respectively. The antioxidant activity data of the extracts were analyzed using a one‐way analysis of variance. Tukey's test was used to compare the antioxidant activities of the extracts. In addition, the Kruskal–Wallis test and Mann–Whitney *U* test with Bonferroni adjustment were used to elucidate the final protective properties of the extract. Pearson's correlation coefficient was also calculated between the different antioxidative properties of the extract and the MTT or NRU test results of RAW264.7 cells exposed to 1.5 μg mL^−1^ AFB_1_. The stepwise regression method was used to investigate relationships between MTT and NRU test results and the antioxidative properties of the extracts (TPC and FRAP). All statistical analyses were interpreted at the 95% confidence level. Results are reported as mean ± SE.

## 3 RESULTS

### 3.1 Antioxidant properties

**FIGURE 1:**
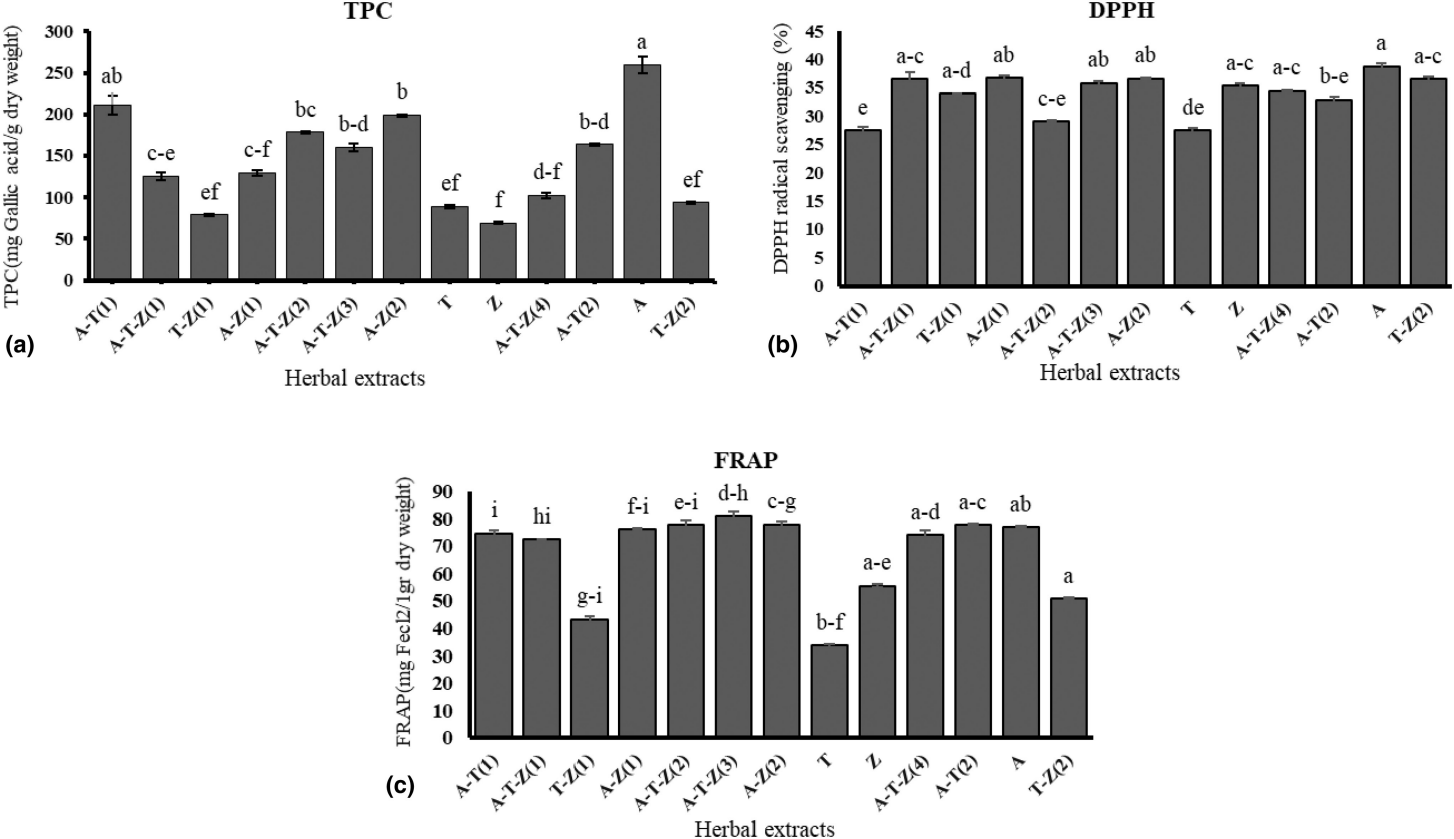
Total phenolic content (a), DPPH^•^ radicals scavenging activity (b), and FRAP assay (c) of the extracts; different letters indicate significant differences at the *p* < .05. Refer to Table 3 for more information regarding the different (multi)herbal extracts used in the assays. A‐T (1) = (1.56 μg mL^−1^ Thyme +0.78 μg mL^−1^ Turmeric), A‐T‐Z (1) = (0.78 μg mL^−1^ Thyme +0.78 μg mL^−1^ Turmeric +1.56 μg mL^−1^ Ginger), T‐Z (1) = (1.56 μg mL^−1^ Turmeric +1.56 μg mL^−1^ Ginger), A‐Z (1) = (0.78 μg mL^−1^ Thyme +3.12 μg mL^−1^ Ginger), A‐T‐Z (2) = (1.56 μg mL^−1^ Thyme +1.56 μg mL^−1^ Turmeric +1.56 μg mL^−1^ Ginger), A‐T‐Z (3) = (1.56 μg mL^−1^ Thyme +0.78 μg mL^−1^ Turmeric +3.12 μg mL^−1^ Ginger), A‐Z (2) = (1.56 μg mL^−1^ Thyme +1.56 μg mL^−1^ Ginger), T = (0.78 μg mL^−1^ Turmeric), Z = (1.56 μg mL^−1^ Ginger), A‐T‐Z (4) = (0.78 μg mL^−1^ Thyme +1.56 μg mL^−1^ Turmeric +3.12 μg mL^−1^ Ginger), A‐T (2) = (0.78 μg mL^−1^ Thyme +1.56 μg mL^−1^ Turmeric), A = (0.78 μg mL^−1^ Thyme), and T‐Z (2) = (0.78 μg mL^−1^ Turmeric +3.12 μg mL^−1^ Ginger).

The highest TPC were observed in Shirazi thyme HAE (A) (259.74 ± 10.49 mg GAE g DM^−1^) and the herbal HAE consisted of Shirazi thyme and turmeric (A‐T (1)) (211.56 ± 11.24 mg GAE g DM^−1^, Figure 1a). Shirazi thyme HAE (A) also showed the highest DPPH• radical scavenging activity (38.81 ± 0.49%, Figure 1b). There was no relation between the DPPH• radical scavenging activity and phenolic content or FRAP test results (Table 3). The FRAP test results also revealed that the antioxidant activity ranged from 70 to 80.78 mg FeCl_2_ g DM^−1^ (Figure 1c). A multi‐herbal extract containing HAEs of ginger, turmeric, and Shirazi thyme (A‐T‐Z (3)) had the highest antioxidant activity (80.78 ± 1.82 mg FeCl_2_ g DM^−1^). It has also been indicated that there was a significantly positive correlation between TPC and MTT results from RAW264.7 cells exposed to 1.5 μg mL^−1^ AFB_1_ (0.721, Table 3). The findings from the regression analysis indicated that the TPC and FRAP assay results of the extracts explained 47 and 22% of the variations observed in the MTT, respectively (Table 4).

**TABLE 3:** Pearson correlation coefficient between different antioxidative properties of the extract and MTT or NRU test results of RAW264.7 cells exposed to 1.5 μg mL^−1^ AFB_1_.

	Rutin	Rosmarinic acid	Coumaric acid	Ferulic acid	Quercetin	Apigenin	Total phenol	FRAP	DPPH	MTT	NRU
Rutin	1	1.000**	.000	.000	.000	.000	.736**	.835**	−.118	.541	.177
Rosmarinic acid		1	.000	.000	.000	.000	.733**	.829**	−.124	.538	.180
Coumaric acid			1	.999**	.000	.000	−0.237	−.096	−.476	.096	.051
Ferulic acid				1	.000	.000	−0.233	−.088	−.467	.109	.055
Quercetin					1	1.000**	−.421	.122	.468	−.378	.522
Apigenin						1	−.416	.125	.466	−.379	.510
Total phenol							1	.700**	.047	.721**	−.291
FRAP								1	.277	.518	−.034
DPPH									1	.035	−.003
MTT										1	−.146
NRU											1

** Correlations are significant at the .01 (2‐tailed).

**TABLE 4:** Regression analyses between the antioxidative properties of the extracts (TPC and FRAP) and MTT assay results of RAW264.7 cells exposed to 1.5 μg mL^−1^ AFB_1_.

Antioxidant properties	Constant	*β*	*R* ^2^ adj	*p*‐Value
Total phenol	1.261**	.001**	.478	.001
FRAP	1.218**	.003**	.229	.001
MTT = 1.261 + 0.001 Phenol				
MTT = 1.218 + 0.003 FRAP				

** Correlations are significant at the .01 (2‐tailed).

**FIGURE 2:**
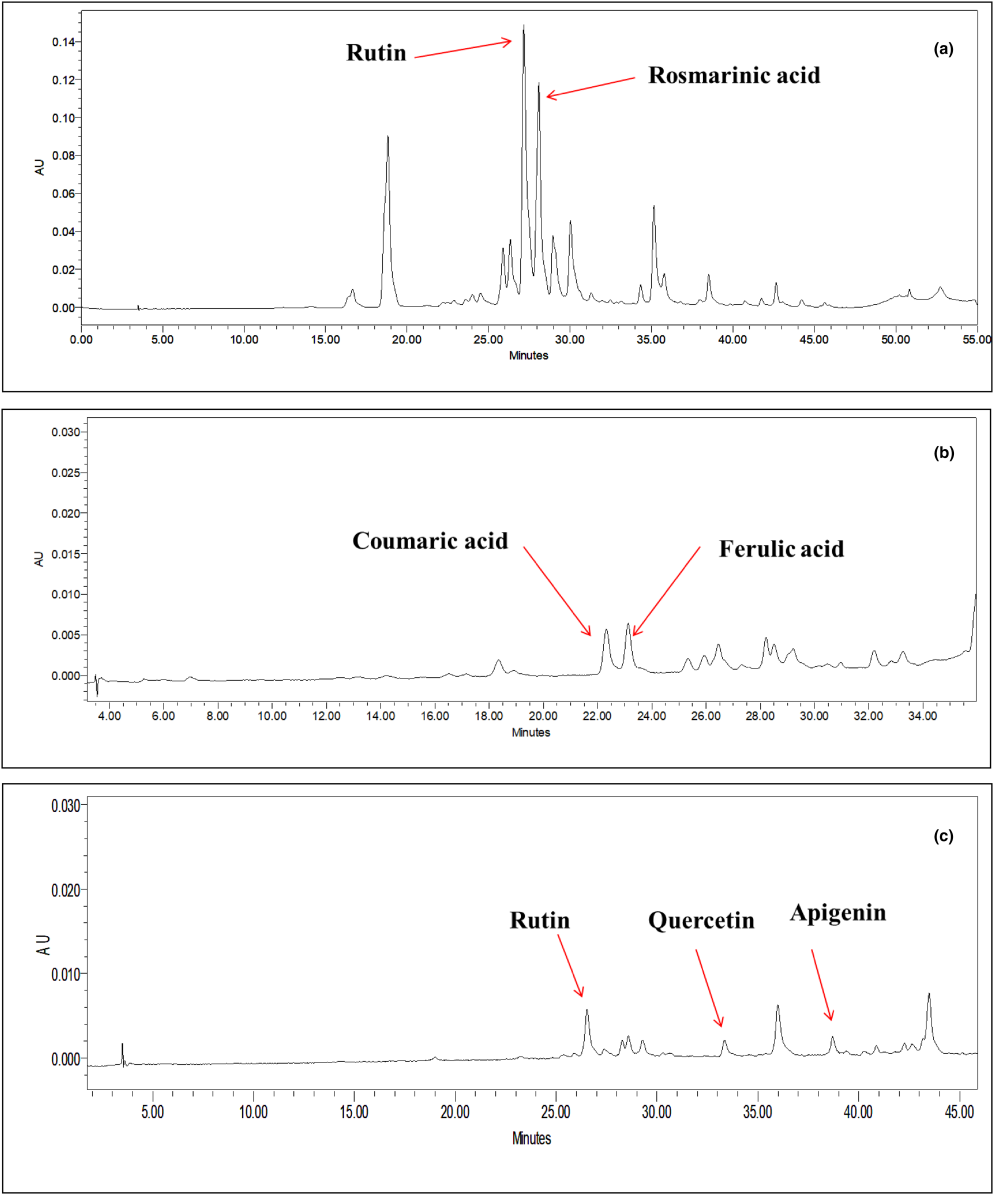
HPLC chromatograms of hydro‐alcoholic extracts of thyme (a), turmeric (b), and ginger (c), depicting the main phenolic compounds of the herbal extracts.

The main phenolic compounds of hydro‐alcoholic extracts of Shirazi thyme, turmeric, and ginger were identified using HPLC (Figure 2). Rutin (87.4 mg g^−1^) and rosmarinic acid (53.8 mg g^−1^) were the main phenolic compounds of Shirazi thyme extract. In comparison, those of turmeric were coumaric acid (1.4 mg g^−1^) and ferulic acid (2.1 mg g^−1^). It has also been revealed that rutin (5.9 mg g^−1^), quercetin (0.7 mg g^−1^), and apigenin (5.1 mg g^−1^) were the abundant phenolic compounds of ginger extract. Additionally, a positive correlation was observed between the rutin content of the HAEs and TPC or FRAP test results (*p* < .001, *r* ^2^ = .736 and .835, respectively). In addition, the rosmarinic acid content of the HAEs was positively correlated with TPC and FRAP results (*p* < .001, *r* ^2^ = .733 and .829, respectively).

The results of stepwise regression analysis showed that the rutin and rosmarinic acid content of the HAEs explained 87% of the observed changes in FRAP (*p* < .05; Table 5).

**TABLE 5:** Regression analysis results between the main phenolic compounds and FRAP assay of the extracts.

Term	Β	SE	Standardized *β*	*t*	Sig.
Constant	45.837	2.79		16.401	0.000
Rutin	× 1016.78 ^7^	38.40 × 10^6^	34.322	4.371	0.001
Rosmarinic acid	× 10−27.18 ^7^	63.73 × 10^6^	−33.490	−4.265	0.002
*F* (2,12) = 41.584, *p*‐value = .00 (*R* ^2^ = .893, *R* ^2^‐adj = .87)					

### 3.2 Determining IC_50_ of AFB_1_ in the RAW264.7 cell line

**FIGURE 3:**
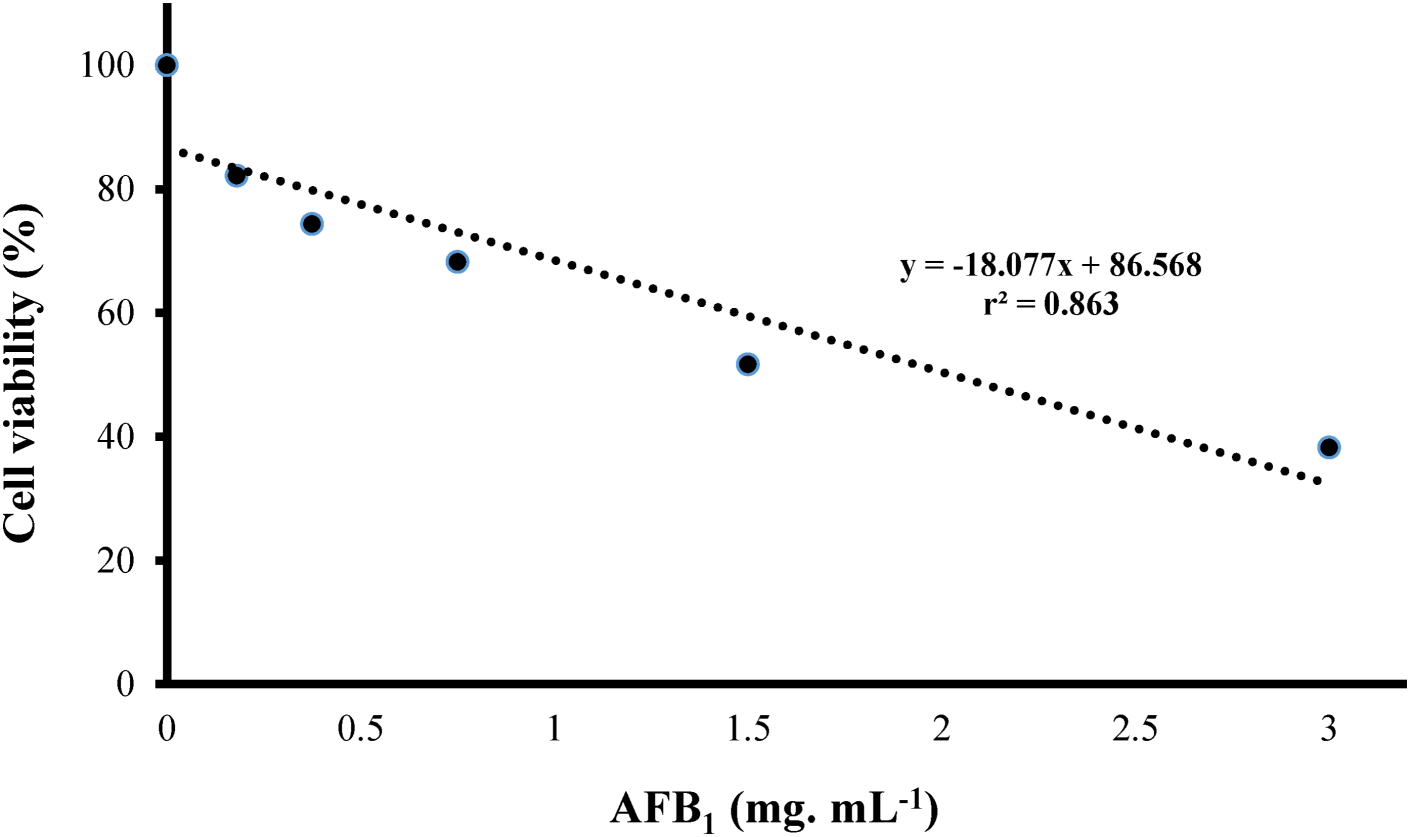
Effect of different concentrations of AFB_1_ on the viability of the RAW264.7 cell line.

According to Figure 3, as the AFB_1_ concentration in the cell culture medium increased, the viability of the RAW264.7 cells decreased (*p* < .05). Accordingly, the IC_50_ of AFB_1_ in the RAW264.7 cell line was 1.5 μg mL^−1^.

### 3.3 Determining the safe concentration of the herbal extracts

**FIGURE 4:**
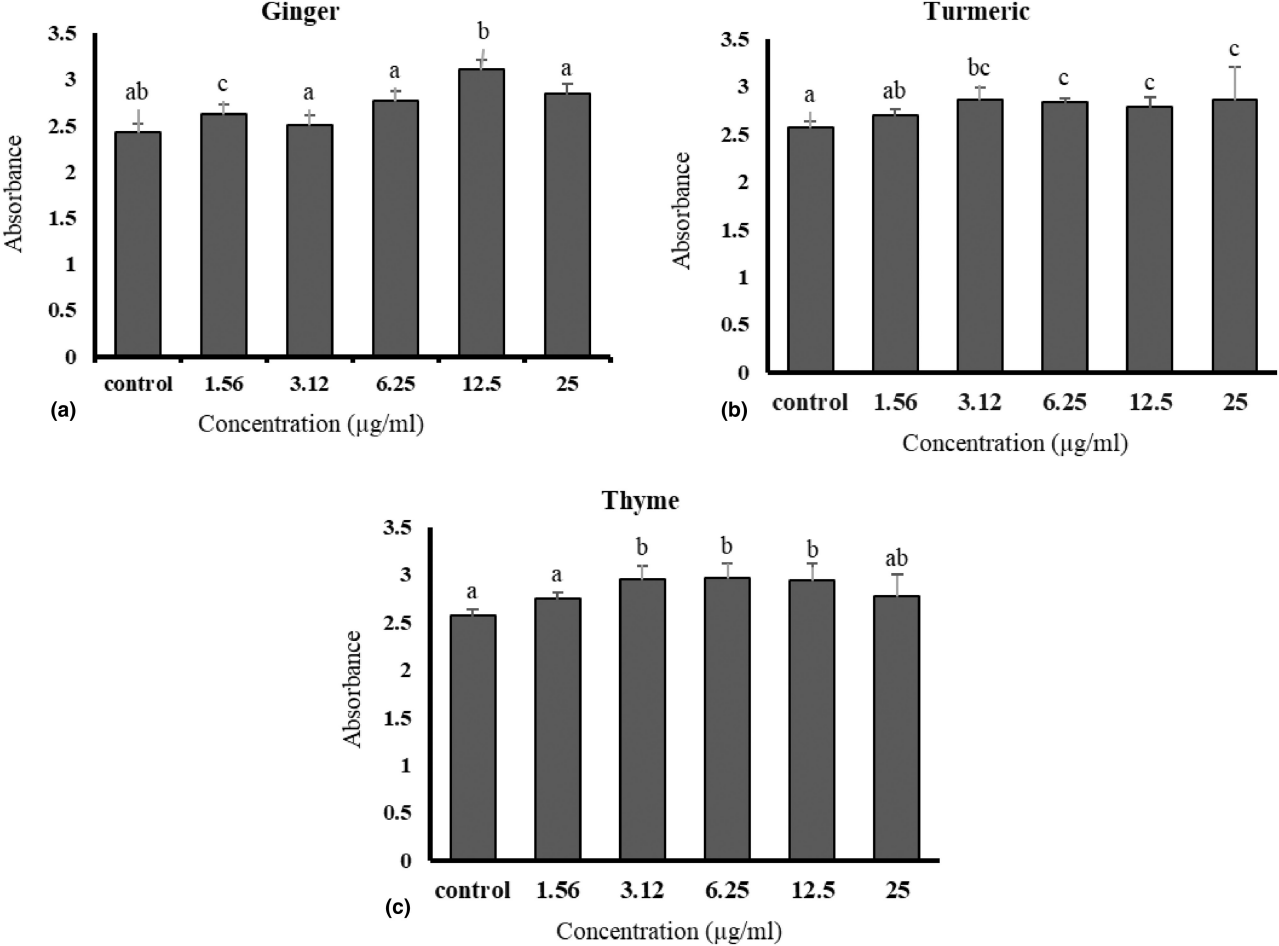
The effect of different concentrations of hydroalcoholic extracts of ginger (a), turmeric (b), and thyme (c) in culture medium on the viability of RAW264.7 cells. Different letters indicate a statistically significant difference (*p* < .05).

**FIGURE 5:**
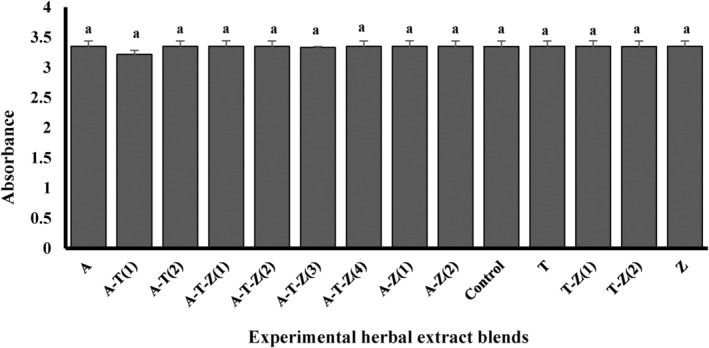
The effect of different concentrations of individual or combinations of hydroalcoholic extracts on the viability of RAW264.7 cells. Different letters indicate a statistically significant difference (*p* < .05). The complete introduction of the treatments is mentioned in Table 1 and the caption of Figure 1.

The results showed that the safe concentration of herbal HAEs was 3.12, 1.56, and 1.56 μg mL^−1^ for ginger, turmeric, and thyme, respectively, without any noticeable effects on cell number or viability (Figure 4). Results also revealed that various combinations of the herbal HAEs did not affect the cells (Figure 5, *p* > .05), indicating their safety to the cell line at the concentration used.

### 3.4 Protective properties of multi‐herbal extracts on AFB_1_‐exposed RAW264.7 cell line

#### 3.4.1 MTT assay

**FIGURE 6:**
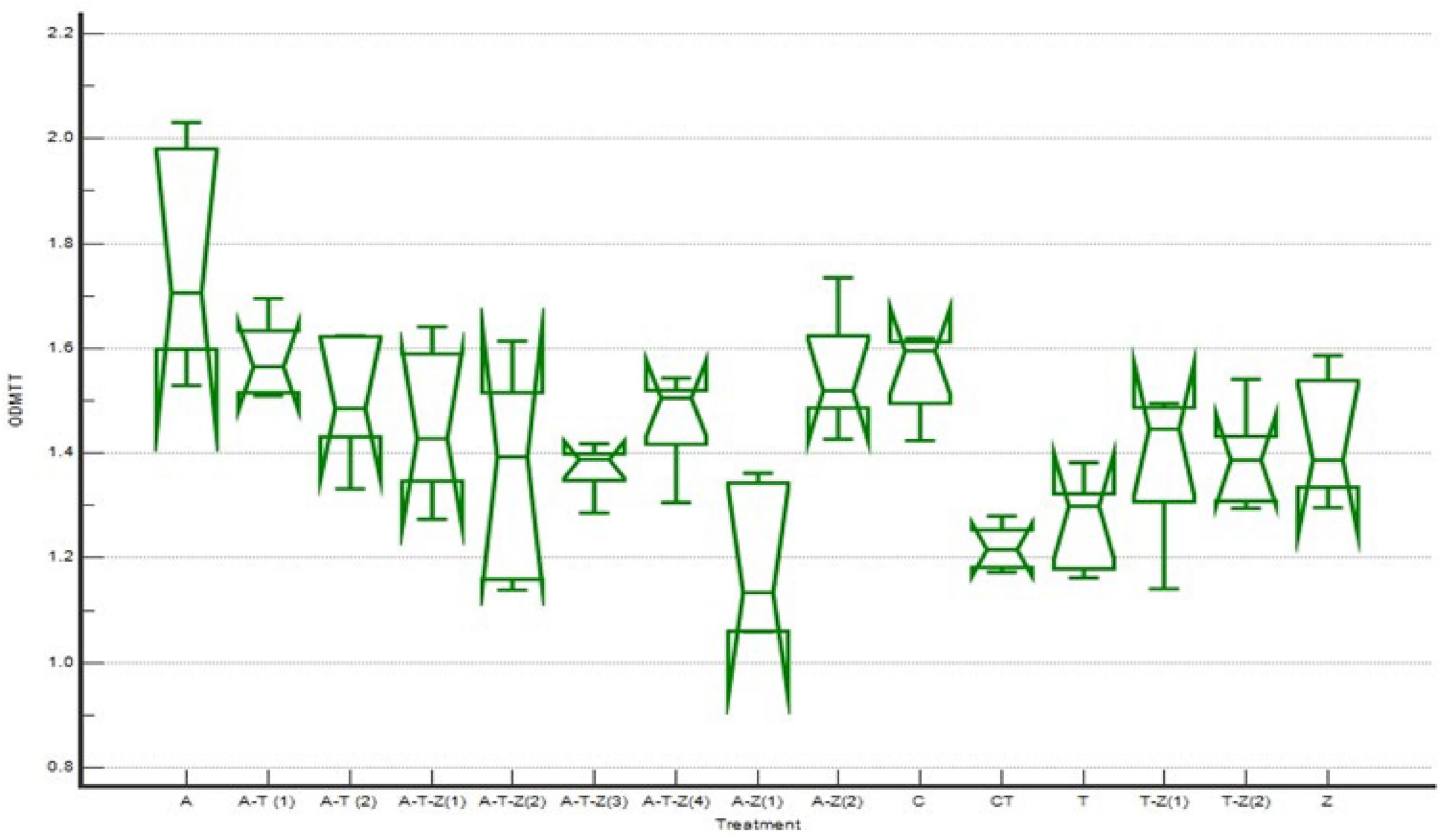
MTT assay results of the protective effect of various combinations of herbal extracts on the RAW264.7 cell line exposed to 1.5 μg mL^−1^ AFB_1_ (the complete introduction of the treatments is mentioned in Table 1 and the caption of Figure 1).

The results of the MTT assay of the cells exposed to 1.5 μg mL^−1^ AFB_1_ revealed that treatments 2, 3, 4, 7, 9, and 15 were followed by treatment one, which showed the highest protective effect on AFB_1_ exposed RAW264.7 cell line (*p* < .05, Table 6), indicating that the extracts protected the cell from the undesirable toxic effects of AFB_1_ to the extent that the MTT results of the groups did not differ from those of the cells exposed to the toxin (*p* > .05, Figure 6).

**TABLE 6:** MTT assay results indicate the protective effect of various combinations of herbal extracts on the RAW264.7 cell line exposed to 1.5 μg mL^−1^ AFB_1_.

Treatment	*n*	Average rank	Different (*p* < .05) from factor NRU
(1) A	5	68.70	(3)(4)(5)(6)(7)(8)(11)(12)(13)(14)(15)
(2) A‐T (1)	5	58.60	(5)(6)(8)(11)(12)(13)(14)(15)
(3) A‐T (2)	5	49.10	(1)(6)(8)(11)(12)
(4) A‐T‐Z (1)	5	41.80	(1)(8)(11)(12)
(5) A‐T‐Z (2)	5	30.60	(1)(2)(8)(9)(10)(11)
(6) A‐T‐Z (3)	5	28.60	(1)(2)(3)(9)(10)
(7) A‐T‐Z (4)	5	43.00	(1)(8)(11)(12)
(8) A‐Z (1)	5	10.80	(1)(2)(3)(4)(5)(7)(9)(10)(13)(14)(15)
(9) A‐Z (2)	5	54.50	(5)(6)(8)(11)(12)(13)(14)
(10) C	5	56.10	(5)(6)(8)(11)(12)(13)(14)
(11) CT	5	10.90	(1)(2)(3)(4)(5)(7)(9)(10)(13)(14)(15)
(12) T	5	16.30	(1)(2)(3)(4)(7)(9)(10)(15)
(13) T‐Z (1)	5	32.20	(1)(2)(8)(9)(10)(11)
(14) T‐Z (2)	5	31.70	(1)(2)(8)(9)(10)(11)
(15) Z	5	37.10	(1)(2)(8)(11)(12)

#### 3.4.2 NRU test

**FIGURE 7:**
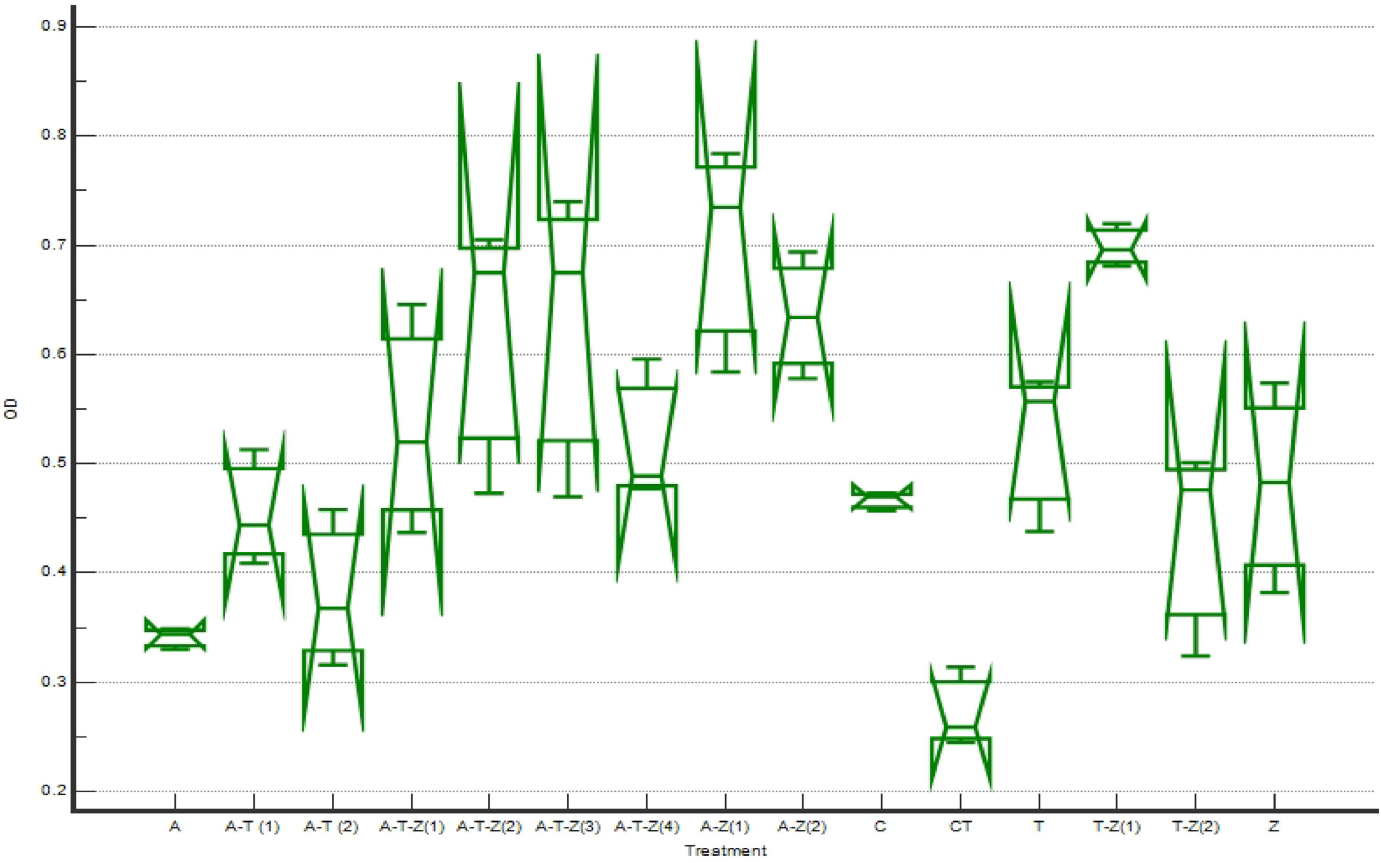
NRU test results of the protective effect of various combinations of herbal extracts on the RAW264.7 cell line exposed to 1.5 μg mL^−1^ AFB_1_ (the complete introduction of the treatments is mentioned in Table 1 and the caption of Figure 1).

According to Figure 7 and Table 7, the best NRU test results were observed in treatments 2 (A‐T (1)), 4 (A‐T‐Z (1)), 7 (A‐T‐Z (4)), 12 (T), 14 (T‐Z (2)), and 15 (Z), indicating that the herbal extracts could prevent the toxic effect of AFB_1_ on the cell membrane.

**TABLE 7:** NRU test results indicate the protective effect of various combinations of herbal extracts on the RAW264.7 cell line exposed to 1.5 μg mL^−1^ AFB_1_.

Treatment	*n*	Average rank	Different (*p* < .05) from factor NRU
(1) A	3	7.00	(4)(5)(6)(7)(8)(9)(12)(13)(15)
(2) A‐T(1)	3	17.00	(5)(6)(8)(9)(11)(13)
(3) A‐T(2)	3	9.67	(4)(5)(6)(7)(8)(9)(12)(13)
(4) A‐T‐Z(1)	3	24.67	(1)(3)(8)(11)(13)
(5) A‐T‐Z(2)	3	32.33	(1)(2)(3)(10)(11)(14)
(6) A‐T‐Z(3)	3	32.67	(1)(2)(3)(10)(11)(14)
(7) A‐T‐Z(4)	3	26.33	(1)(3)(8)(11)(13)
(8) A‐Z(1)	3	40.00	(1)(2)(3)(4)(7)(10)(11)(12)(14)(15)
(9) A‐Z(2)	3	34.67	(1)(2)(3)(10)(11)(14)(15)
(10) C	3	17.33	(5)(6)(8)(9)(11)(13)
(11) CT	3	2.00	(2)(4)(5)(6)(7)(8)(9)(10)(12)(13)(14)(15)
(12) T	3	23.67	(1)(3)(8)(11)(13)
(13) T‐Z(1)	3	40.00	(1)(2)(3)(4)(7)(10)(11)(12)(14)(15)
(14) T‐Z(2)	3	17.00	(5)(6)(8)(9)(11)(13)
(15) Z	3	20.67	(1)(8)(9)(11)(13)

### 3.5 Introducing a better combination of herbal extracts using MTT and NRU test results

The overall protective capability of different experimental herbal blends on the RAW264.7 cell line exposed to 1.5 μg mL^−1^ AFB_1_ was ranked according to their MTT and NRU assay results from 15 (the highest protection rank), to 1 (the lowest protection rank) to consist of cell functionality in terms of cell metabolism and cell membrane integrity (Table 8). Accordingly, four experimental treatments, including treatments 2 (A‐T (1)), 4 (A‐T‐Z (1)), 7 (A‐T‐Z (4)), and 15 (Z), showed the highest overall ranking score, indicating their proportionally better protective properties in maintaining the vitality and/or functionality of the cells exposed to the IC_50_ of AFB_1_. Practically, treatment A‐T (1), composed of 1.56 μg mL^−1^ of thyme extract and 0.78 μg mL^−1^ of turmeric extract, seemed the best choice regarding efficiency and cost‐effectiveness.

**TABLE 8:** The overall protective capability of different experimental herbal blends on the RAW264.7 cell line exposed to 1.5 μg mL^−1^ AFB_1_.

Group	Experimental treatments	MTT	NRU	Ranking
1	A	15	2	17
2	(1)A‐T	14	6	20
3	(2)A‐T	12	3	15
4	(1)A‐T‐Z	10	10	20
5	(2)A‐T‐Z	4	11	15
6	(3)A‐T‐Z	5	12	17
7	(4)A‐T‐Z	11	8	19
8	(1)A‐Z	1	15	16
9	(2)A‐Z	13	13	26
10	(blank) C	3	4	7
11	(control) CT	8	1	9
12	T	2	9	11
13	(1)T‐Z	6	14	20
14	(2)T‐Z	7	5	12
15	Z	9	7	16

*Note*: The complete introduction of the treatments is mentioned in Table 1 and the caption of Figure 1.

### 3.6 Apoptotic and necrotic cells using fluorescence microscopy

**FIGURE 8:**
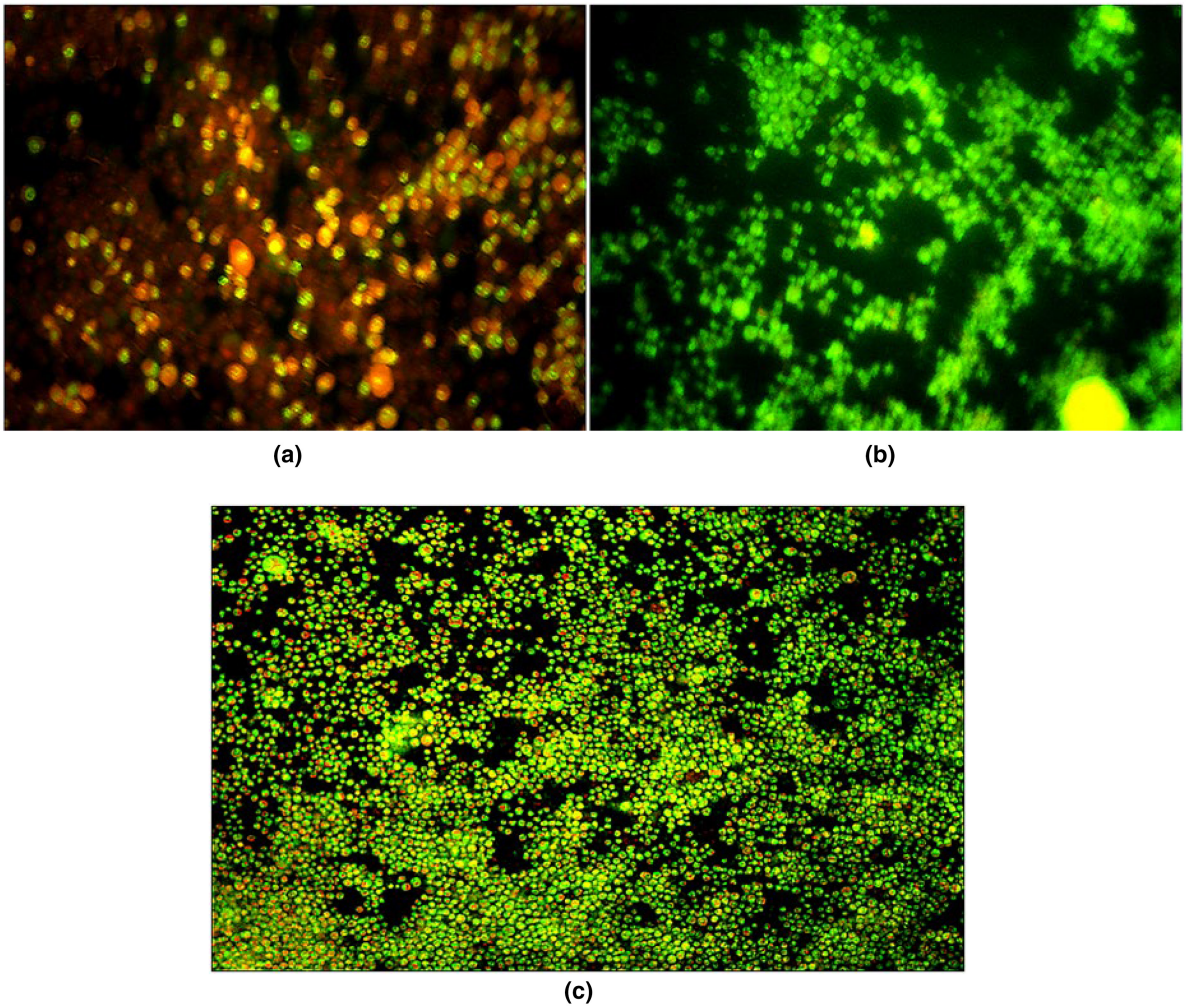
(a) Fluorescence microscopy view of RAW264.7 cells exposed to 1.5 μg mL^−1^ AFB_1_. The cells were stained with Acridine Orange and Propidium Iodide. Red stain indicates apoptotic cells and green stain points to live cells; (b) Live and healthy cells; and (c) Fluorescence microscopy view of RAW264.7 cells exposed to 1.5 μg mL^−1^ AFB_1_ and A‐T (1) (magnification at 40×).

Fluorescent microscopy observation (Figure 8) revealed that the control group (RAW264.7 cells exposed to 1.5 μg mL^−1^ AFB_1_) showed the highest number of secondary apoptotic cells (Figure 8a). Live and healthy cells were observed to be uniformly green (Figure 8b). In the best‐ranked plant extract, A‐T (1), a decrease in the severity of secondary apoptotic cells was evident in comparison to the control group (Figure 8c).

## 4 DISCUSSION

The hydro‐alcoholic extract of Shirazi thyme showed the highest TPC (259.74 ± 10.49 mg GAE g DM^−1^), followed by a multiherbal extract consisting of Shirazi thyme and turmeric (211.56 ± 11.24 mg GAE g DM^−1^). A significantly positive correlation was observed between the TPC and MTT assays of the cells exposed to 1.5 μg mL^−1^ AFB_1_. The TPC of Shirazi thyme in the present study was close to the values previously reported in the literature (Bazargani‐Gilani et al., 2014). Bahrami‐Karkavandi et al. (2011) reported that with an increase in the concentration of phenolic compounds, the number of hydroxyl groups in the reaction medium increases, which might result in improved free radical hydrogenation and higher antioxidative properties of the extracts. Attaran et al. (2018) reported that Shirazi thyme essential oil intake would significantly affect oxidative/antioxidant stress indices, including lipid peroxidation (LP) and glutathione (GSH) and antioxidant enzyme activity (e.g., glutathione‐S‐transferase (GST)), in a rat model against iron oxide nanoparticle hepatotoxicity. The free radical scavenging activity of the methanolic extract of Shirazi thyme was greater than that of ascorbic acid (Souri et al., 2008). In another study, Bazargani‐Gilani et al. (2014) stated that Shirazi thyme essential oil showed better antioxidative activity and higher TPC (262.52 mg/g) than Iranian pomegranate juice. Investigating the free radical inhibitory activity of essential oils and different fractions of methanol extracts of Shirazi thyme, sage (*Salvia officinalis*), rosemary (*Rosmarinus officinalis*), Khalivash (*Mentha pulegium*), and cinnamon (*Cinnamomum zeylanicum*), Hosseini et al. (2012) found that the highest free radical inhibitory activity belonged to Shirazi thyme essential oil. A higher antioxidative activity of Shirazi thyme essential oil was attributed to its higher phenolic contents, including thymol and carvacrol (Ruberto & Baratta, 2000).

Curcumin has been identified as an anti‐inflammatory and antioxidant compound (Mele, 2019). Structurally, its higher methoxylation capacity and lower hydrogenation rate increased free radical scavenging activity. It has been shown that the structure probably enables curcumin to have anti‐cancer, anti‐inflammatory, and antioxidant effects (Devassy et al., 2015).

In the present research, the TPC of the HAE of turmeric was 88.55 ± 2.12 (mg GAE g DM^−1^), while Sepahpour et al. (2018) reported that the TPC of the acetone extract of turmeric was 221.7 ± 0.9 (mg GAE g DM^−1^), which might be attributable to the solvent used for extraction. Samiei et al. (2018) reported that turmeric essential oil contained 8.40 ± 0.50 (mg GAE g DM^−1^) TPC. Meanwhile, an ethanolic extract of turmeric contained 92.45 mg GAE g DM^−1^ TPC (Kaur & Kapoor, 2002). Aydin and Kadioglu (2022) found that the TPC of ethyl acetate extract and dichloromethane extract of turmeric was 175.74 ± 0.050 and 93.66 ± 0.013 μg GAE mL^−1^, respectively. Farahpour et al. (2014) reported that the average TPC of HAE of turmeric rhizome was 15.48 ± 0.3 (mg GAE g DM^−1^). It has been indicated that curcumin is a potent antioxidant that reduces the formation of ROS and improves the body's antioxidant defense system (Pyun et al., 2014).

Ginger contains polyphenol compounds with numerous antioxidative activities (Yudthavorasit et al., 2014). In the present study, the HAE of ginger contained 68.87 ± 1.27 (mg GAE g DM^−1^) TPC. However, the TPC for the acetone extract of ginger was 35.67 ± 1.89 (mg GAE g DM^−1^) (Ahmadtabar Kalebasti et al., 2019). Investigating the antioxidant properties of ginger and turmeric rhizomes, Erdogan and Erbas (2021) reported that the TPC of ethanolic extracts of ginger and turmeric were 48.56 ± 1.64 and 82.47 ± 2.70 mg GAE g DM^−1^, respectively. In another study, EL‐Ghorab et al. (2010) found that the maximum TPC of methanol and hexane‐extracted fresh ginger was 5.70 and 3.96 (mg GAE g DM^−1^), respectively. While Tohma et al. (2017) reported that the TPC of the ethanolic extract of ginger was 137.5 (μg GAE mg DM^−1^).

DPPH• is widely used to evaluate the free radical scavenging ability of herbal extracts or antioxidant compounds (Sridhar & Charles, 2019). The DPPH• activity of Shirazi thyme extract (38.81%) was similar to the values that have already been reported (Mazarie et al., 2018). Shirazi thyme extract showed a considerable ability to donate electrons to free radicals, converting them into more stable, non‐reactive compounds (Keramat et al., 2018).

It has been shown that biomolecules are oxidized by reacting metals with ROS through the Fenton reaction (Gulcin, 2009). The ferric‐reducing power of the HAEs ranged from 70 to 80.78 mg FeCl_2_ g DM^−1^. The results also revealed that the herbal extract of ginger, turmeric, and Shirazi thyme showed the highest FRAP value, 80.78 ± 1.82 mg FeCl_2_ g DM^−1^. Also, it is worth mentioning that the antioxidant power of herbal extracts improves with increasing the polarity of the solvent used for extraction since this fraction is mainly composed of phenolic compounds (Ara & Nur, 2009), which is in agreement with our finding regarding the significantly positive correlation between TPC and FRAP or MTT assay results.

Similar to our results, Nazaryanpour and Nejad Ebrahimi (2020) reported three flavonoid glycosides, including apigenin 7‐O‐glucoside, luteolin 7‐O‐glucopyranoside, and luteolin 7‐O‐rutinoside, along with rosmarinic acid, oleanolic acid, and ursolic acid, in the methanolic extract of Shirazi thyme. It has also been found that HAE of *Thymus caramanicus* shoots contained flavonoids (i.e., luteolin, rutin, and quercetin) and phenolic acids (i.e., rosmarinic, and caffeic acids) (Honari et al., 2018). Wang et al. (2004) also reported that *Thymus vulgaris* L. contained 4.5–8.7 mg g^−1^ of rosmarinic acid. Similarly, Hyytia et al. (1999) reported that turmeric extract contained ferulic and protocatechuic acids. Meanwhile, Tohma et al. (2017) found that pyrogallol, p‐hydroxybenzoic acid, ferulic acid, and p‐coumaric acid were the most abundant phenolic compounds in ethanolic and aqueous extracts of ginger.

Results revealed that viability and vitality of the macrophage cell line were significantly affected by AFB_1_, with an IC_50_ of 1.5 ± 0.2 μg mL^−1^. Similar to our results, Zimmermann et al. (2014) found that the viability of lymphocyte‐rich mononuclear cells in broiler chickens was considerably reduced in a time and dose‐dependent manner following exposure to 10 μg mL^−1^ AFB_1_. They also reported that ROS formation significantly increased after exposure to AFB_1_, which might subsequently affect lymphocyte viability. It has been shown that AFB_1_ and aflatoxin M_1_ (0.01–1 μg mL^−1^) significantly inhibited human Caco‐2 cell growth. The toxins also decrease cell viability while increasing lactate dehydrogenase release and causing genetic damage in a time and dose‐dependent manner (Zhang et al., 2015). Consistent with our results, Moradi et al. (2015) reported that the viability of normal human breast epithelial cells decreases as the AFB_1_ concentration increases in the medium.

Plants are rich in bioactive compounds, classified into primary and secondary metabolites depending on their roles in plant life (Sharma et al., 2019). Plant secondary metabolites can ameliorate the toxic and genotoxic effects of mycotoxins by preventing/reducing free radical formation (Wu et al., 2017) or by inducing xenobiotic detoxification and biotransformation pathways (Wu et al., 2017). They might be additives to prevent fungal growth and aflatoxin (AF) contamination in food/feed (Makhuvele et al., 2020).

Our results showed that (multi‐herbal) extracts of ginger, turmeric, and Shirazi thyme were able to mitigate the toxicity of AFB_1_ in RAW264.7 macrophage cells. The highest protection efficiency regarding MTT or NRU assay results was observed in the cells cultured on AFB_1_ contaminated media with a hyrdroalcoholic extract of Shirazi thyme (0.78 μg mL^−1^ (A)). While the highest NRU assay results belonged to a combination of three herbal extracts, including 0.78 μg mL^−1^ Shirazi thyme extract, 0.78 μg mL^−1^ turmeric extract, and 1.56 μg mL^−1^ ginger extract (A‐T‐Z (1)). Regarding overall protection efficiency, the combination of 1.56 μg mL^−1^ Shirazi thyme extract and 0.78 μg mL^−1^ turmeric extract (A‐T (1)) showed promising results. Ponzilacqua et al. (2019) examined the AFB_1_ degradation activity of rosemary (*Rosmarinus officinalis*), oregano (*Origanum vulgare*), araca (*Psidium cattleianum*), and sweet passion fruit (*Passiflora alata*) extracts. They found that rosemary extract had the highest activity of AFB_1_ degradation (49.0%–60.3%) during 24–48 h, followed by oregano (30.7%–38.3%) after 48 h. Regarding ginger extract, similar to our results, Vipin et al. (2017) reported that pretreatment of HepG2 cells with ginger extract significantly inhibited intracellular ROS production, DNA double‐strand breaks, and, therefore, the cytotoxicity of AFB_1_. Iram et al. (2016) found that sweet basil (*Ocimum basilicum*) leaf extracts degraded AFB_1_ and AFB_2_ (90.4% and 88.6%, respectively). They also reported that the extracts of sweet basil leaves considerably decreased aflatoxigenic fungal isolates (82%–87%) growth. Lower AFB_1_ toxicity to the cells might also be due to the removal of the double bond in the furan ring and changes in the lactone ring of AFs by herbal extracts since it has been shown that the double bond in the furan ring of the AFB_1_ molecule is the main cause of its toxic and carcinogenic activity (Vijayanandraj et al., 2014; Wang et al., 2011). The exact mechanisms behind the modulating molecular structure, and therefore, the toxicity of mycotoxins in herbal extracts, require further clarification in the future. For instance, it has been shown that compounds such as alkaloids might be actively involved in such molecular changes in toxins (Vijayanandraj et al., 2014). Pauletto et al. (2020) evaluated the potential anti‐ AFB_1_ (3.6 μM) activity of a curcumin compound (2.5, 5, and 10 μM) in bovine fetal hepatocytes (BFH12). They found that curcumin reduced AFB_1_‐induced cell death by ca. 30% via its antioxidative and anti‐inflammatory activities. Mathuria and Verma (2007) reported that turmeric extracts/curcumin (1–100 μg mL^−1^) significantly reduced AFB_1_‐induced hemolysis (0.5–2.0 μg mL^−1^), in vitro. Similarly, we found that the hydro‐alcoholic extract of turmeric in combination with Shirazi thyme extract improved cell viability and vitality when exposed to 1.5 μg mL^−1^ AFB_1_.

## 5 CONCLUSION

In conclusion, our results revealed that multiherbal hydro‐alcoholic extracts including Shirazi thyme and turmeric, i.e. A‐T (1), and Shirazi thyme, turmeric, and ginger, i.e. A‐T‐Z (1), showed the highest protective effect against 1.5 μg mL^−1^ AFB_1_ exposure of the RAW264.7 cell line. Such protective potency might be attributable to the antioxidative properties originated from the considerably higher phenolic compounds of those extracts. The compounds might involve in inhibition and or scavenging of free radicals released during the cell AFB_1_ exposure or even their ability to modify the chemical structure of the toxin and rendering it less toxic. However, the exact mechanism of their bioactive properties against the toxins requires further elucidation in future studies.

## AUTHOR CONTRIBUTIONS

**Nina Nazdar:** Data curation (equal); investigation (equal); writing – original draft (equal). **Ahmad Imani:** Conceptualization (equal); formal analysis (equal); funding acquisition (equal); project administration (equal); writing – review and editing (equal). **Seyyed Meysam Abtahi Froushani:** Methodology (supporting); supervision (supporting). **Mohsen Farzaneh:** Formal analysis (supporting); methodology (supporting); validation (supporting). **Kourosh Sarvi Moghanlou:** Methodology (supporting); validation (supporting).

## FUNDING INFORMATION

The research was supported by the Research Council of Urmia University under grant No. 10148 as a part of the PhD thesis of Nina Nazdar under the co‐supervision of Drs. Ahmad Imani and Seyyed Meysam Abtahi Froushani.

## CONFLICT OF INTEREST STATEMENT

Authors declare that they do not have any conflicts of interest.

## DECLARATIONS

In the present study, only the mouse macrophage RAW264.7 cell line was used, and no observations were made on any animal subjects.

## Data Availability

All data generated or analyzed during this study are included in this published article.

## Footnotes

^1^
Photodiode Array: A Common type of detector in HPLC.
